# Predictors of silicosis and variation in prevalence across mines among employed gold miners in South Africa

**DOI:** 10.1186/s12889-020-08876-2

**Published:** 2020-06-01

**Authors:** Dave Knight, Rodney Ehrlich, Annibale Cois, Katherine Fielding, Alison D. Grant, Gavin Churchyard

**Affiliations:** 1 International SOS, Singapore, Singapore; 2 Centre for Environmental and Occupational Health Research, School of Public Health and Family Medicine, University of Cape Town, PO Box 43915, Scarborough, Cape Town, 7975 South Africa; 3 Burden of Disease Research Unit, South African Medical Research Council, Parow Valley, South Africa; 4 Division of Epidemiology and Biostatistics, School of Public Health and Family Medicine, University of Cape Town, Cape Town, South Africa; 5 School of Public Health, University of the Witwatersrand, Johannesburg, South Africa; 6 London School of Hygiene and Tropical Medicine, London, UK; 7 Africa Health Research Institute, School of Nursing and Public Health, University of KwaZulu-Natal, Durban, South Africa; 8 The Aurum Institute, Johannesburg, South Africa

**Keywords:** Silicosis, Tuberculosis, Gold mining, South Africa

## Abstract

**Background:**

The stated intention to eliminate silicosis from the South African goldmining industry as well as current programmes to find and compensate ex-miners with silicosis require an understanding of variation in silicosis prevalence across the industry. We aimed to identify the predictors of radiological silicosis in a large sample of working miners across gold mines in South Africa.

**Methods:**

Routine surveillance chest radiographs were collected from 15 goldmine “clusters” in a baseline survey undertaken in preparation for a separate tuberculosis isoniazid prophylaxis trial. All images were read for silicosis by a health professional experienced in using the International Labour Organisation (ILO) classification. Profusion thresholds of > 1/0 and > 1/1 were used. Demographic and occupational information was obtained by questionnaire. Predictors of silicosis were examined in a multivariable logistic regression model, including age, gender, racial ascription, country of origin, years since starting mine employment, mine shaft, skill category, underground work status and tuberculosis.

**Results:**

The crude silicosis prevalence at ILO > 1/1 was 3.8% [95% confidence interval (CI) 3.5–4.1%]. The range across mine shafts was 0.8–6.9%. After adjustment for covariates, the interquartile range across shafts was reduced from 2.4 to 1.2%. Black miners [adjusted odds ratio (aOR) 2.8; 95% CI 1.1–7.2] and miners in full-time underground work (aOR 2.1; 95% CI 1.3–3.4) had substantially elevated odds of silicosis, while workers from Mozambique had lower odds (aOR 0.54; 95% CI 0.38–0.77). Silicosis odds rose sharply with both age and years since starting in the industry (p for linear trend < 0.005), with 95.5% of affected miners having > 15 years since first exposure and 2.2% < 10 years.

**Conclusions:**

In surveillance of silicosis in working gold miners time since first exposure remains a powerful predictor. Age appears to be an independent predictor, while the detection of radiological silicosis in short-service miners requires attention. Public risk reporting by mines should include factors bearing on silicosis prevalence, specifically dust concentrations, with independent verification. Studies of silicosis and tuberculosis in ex-miners are needed, supported by an accessible electronic database of the relevant medical and dust exposure records of all gold miners.

## Background

Silicosis, fibrosis of the lung due to inhalation of silica containing dust, remains a disease of the modern age [1]. Silicosis continues to be reported from traditional extractive and industrial operations in Brazil [2], China [3], India [4] and Zambia [5], among many others. Severe silicosis has appeared in new industrial settings, specifically the fabrication of engineered stone [6] and denim apparel sandblasting [7].

In South Africa, silicosis is currently at the centre of several major public health and legal responses to the serious epidemic of occupational lung disease in the South African gold mining industry uncovered in studies published from the late 1990s onwards [8–14]. In 2003, with a recommitment in 2013, mining employers, labour and the government agreed on a voluntary target of eliminating silicosis in the population of new gold miners starting employment from 2008 onwards, such that after 2013 no new cases of silicosis would occur [15]. In 2012 the Southern African Development Community (SADC) issued a Declaration calling for control of silica dust exposure and surveillance of silicosis as among the elements needed to control the mining related tuberculosis epidemic [16].

An unprecedented class action suit against a number of gold mining companies for silicosis and mining related tuberculosis was settled in 2019 for 5 billion ZAR (approx. US$350 million) [17]. The Tshiamiso Trust has been formed to administer the examination and compensation of eligible claimants over the next 12 years [17, 18]. In parallel, the state miners’ compensation agency has launched a large-scale initiative to clear a huge backlog of claims, as well as find, examine and certify miners who may be eligible for compensation for silicosis or silico-tuberculosis under the mining compensation statute [19]. The number of eligible claimants under the above schemes, statutory and litigation based, is unknown but estimates include hundreds of thousands [19, 20].

Finally, in an effort to mitigate the epidemic of tuberculosis among miners and ex-miners, the Global Fund to Fight HIV, Tuberculosis and Malaria is funding a $300 million programme to establish “infrastructure, processes and systems” for the screening and treatment of miners, ex-miners, and their families for tuberculosis in miner communities in ten Southern Africa countries, which includes screening for silicosis [21].

These initiatives range across the public health hierarchy of prevention which starts with primary prevention (reduction of respirable silica levels in mining operations), but includes also secondary prevention (surveillance, early detection and treatment/management of tuberculosis and silicotuberculosis among working and former gold miners) and tertiary prevention (compensation and social security for affected miners). The implementation of these initiatives across a changing industry needs a thorough epidemiological understanding of the factors influencing the prevalence of silicosis.

None of the studies cited earlier investigated silicosis prevalence across the industry as a whole. However, conduct of a large tuberculosis prevention trial using isoniazid prophylaxis between 2006 and 2011 (the *Thibela TB* study [22, 23] provided us with an opportunity to study the prevalence of silicosis among working gold miners across a number of gold mines. Overall silicosis prevalence findings from this study have been published [24], revealing that there had been no decline in silicosis prevalence, once age was controlled for, from that of a study carried out in one company 25 years previously [25].

This report takes this analysis further than overall prevalence, by investigating two questions relevant to the prevention of silicosis as defined above. First, how does silicosis prevalence *vary* across mine shafts and geographic regions? Second, of the surveillance information that is generally available to interested parties, what are the *occupational and demographic predictors* of silicosis. This information should in turn inform the activities of mining companies, government agencies, compensation trusts, labour organisations and regional groupings such as SADC regarding future surveillance of silicosis, and evaluation of progress towards control and elimination of the disease.

## Methods

### Selection of participants

The Thibela TB (‘Prevent TB’) study was a cluster randomised trial of isoniazid preventive treatment conducted in 16 mine shafts, organised into 15 “clusters”.^1^ These shafts were operated by three independent gold mining companies in three geographically distinct mining areas of South Africa. Inclusion criteria for clusters were a workforce minimum of 1000 miners and an expected mine lifespan of least 10 years. Design, and methods of the study (including development of a survey questionnaire from which data for this study were derived) have been previously described [22, 23] and the results of the Thibela TB trial have been published [23].

In the baseline phase of the Thibela TB study, a random sample of approximately 1000 miners in each cluster (a subset of the total workforce) were surveyed, staggered over the period 2006–2009. Enrolment to the baseline survey was in two stages: (1) a random sample of miners were invited to attend the study centre after their shift; and (2) attendees who gave informed consent to participate in the study were enrolled. A questionnaire completed in the baseline survey collected information on age, gender, racial ascription, country of origin, years since starting mine employment, mine shaft, current occupational location (underground or surface) and skill category (skilled or unskilled). At the request of labour unions, human immunodeficiency virus (HIV) testing was not done as part of the study.

The most recent routine chest radiograph from the mine medical services as part of the annual screening of miners was retrieved. These were dated between June 2004 and February 2009 (median interval 5.3 months before the baseline interview, maximum 21 months).

Radiographs, almost all digital, were read for silicosis according to the International Labour Organisation (ILO) classification [26] by a nursing health professional with 30 years of experience in screening radiographs for silicosis and tuberculosis, and whose previous accuracy has been validated against autopsy findings [27]. The baseline survey and methods of the silicosis study have been previously described in detail [24].

### Statistical analysis

Analysis for the current report was based on silicosis defined by radiological profusion of ILO grade ≥ 1/1 and ≥ 1/0 in separate analyses. Tuberculosis was first categorised as self-reported treatment for tuberculosis (“past” or “current”) or radiological evidence of past or current disease. These metrics were combined into a single tuberculosis variable (self-reported or radiological evidence of tuberculosis or both). Multivariate logistic regression was carried out with individual radiological silicosis status as the dependent variable and all demographic and occupational covariates described above as the independent variables (see also Table 1). Unadjusted and fully adjusted models were fitted. A separate model was run substituting company (A, B and C) and geographic region (Carletonville, Orkney and Welkom) for the 15 clusters. Further models were run adjusting for tuberculosis using the metric described above. Tests for linear trend were used for the associations with age and years since first exposure.

**Table 1:** Prevalence of silicosis by demographic and occupational variables (*N* = 14,221)

			Profusion score ≥ 1/1		Profusion score ≥ 1/0	
		N (column %)	n	Crude prevalence %	n	Crude prevalence %
**Overall**		14,221 (100)	535	3.76	730	5.13
**Cluster**	1	1138 (8.00)	39	3.43	54	4.74
	2	859 (6.04)	25	2.91	31	3.61
	3	353 (2.48)	10	2.83	11	3.12
	4	922 (6.48)	48	5.21	61	6.61
	5	946 (6.65)	32	3.38	45	4.76
	6	1006 (7.07)	70	6.96	81	8.05
	7	948 (6.67)	26	2.74	40	4.22
	8	960 (6.75)	13	1.35	22	2.29
	9	659 (4.63)	24	3.64	36	5.46
	10	1085 (7.63)	71	6.54	93	8.57
	11	1004 (7.06)	57	5.68	76	7.57
	12	1140 (8.02)	32	2.80	59	5.17
	13	1013 (7.12)	9	0.89	18	1.78
	14	1151 (8.09)	34	2.95	44	3.82
	15	1037 (7.29)	45	4.34	59	5.70
**Region**	Carletonville	6726 (47.30)	209	3.11	307	4.56
	Orkney	3104 (21.83)	150	4.83	192	6.18
	Welkom	4391 (30.88)	176	4.00	231	5.26
**Company**	A	5395 (37.94)	216	4.00	295	5.47
	B	3779 (26.57)	98	2.59	140	3.70
	C	5047 (35.49)	221	4.38	295	5.84
**Gender**	Male	13,894 (97.70)	534	3.84	728	5.24
	Female	327 (2.30)	2	0.61	2	0.61
**Age (yrs)**	18–34	3361 (23.63)	9	0.27	14	0.42
	35–39	2062 (14.50)	17	0.82	30	1.45
	40–44	2998 (21.08)	72	2.40	103	3.45
	45–49	2965 (20.85)	179	6.03	253	8.53
	50+	2835 (19.94)	258	9.10	330	11.64
**Racial ascription**	Black	13,790 (96.97)	530	3.84	721	5.23
	Other	431 (3.03)	5	1.16	8	1.86
**Country of origin**	South Africa	8062 (56.69)	261	3.24	364	4.51
	Lesotho	3510 (24.68)	192	5.47	256	7.29
	Swaziland	565 (3.97)	21	3.71	26	4.60
	Mozambique	1853 (13.03)	44	2.37	64	3.45
	Botswana	223 (1.57)	16	7.17	20	8.97
	Other	8 (0.06)	0	0.00	0	0.00
**Years since entry into industry**	0-4^a^	2314 (16.27)	5	0.22	10	0.43
	5–9	1276 (8.97)	7	0.55	11	0.86
	10–14	1724 (12.12)	12	0.70	15	0.87
	15–19	1956 (13.75)	39	1.99	61	3.12
	20–24	2479 (17.43)	90	3.63	120	4.84
	25–29	2113 (14.86)	143	6.77	202	9.56
	30+	2359 (16.59)	239	10.13	311	13.18
**Skill level**	Unskilled	12,981 (91.28)	506	3.90	694	5.35
	Skilled/Official	1240 (8.72)	28	2.26	35	2.82
**Underground work**	Full time	13,245 (93.14)	515	3.89	705	5.32
	None/part time	976 (6.86)	20	2.05	25	2.56
**Tuberculosis**	On CXR and/or self-reported treatment	3046 (21.42)	194	6.37	241	7.91
	Neither	11,175 (78.58)	341	3.05	488	4.37

*CXR* Chest radiograph, *N* number of subjects in each category, *n* number of cases of silicosis

^a^Including 202 subjects with “0” years in the industry recorded on the questionnaire

To further explore the existence of an exposure threshold for silicosis, the model was refitted with years since first exposure as a continuous variable allowing a non-linear relationship with silicosis risk, by means of a generalised additive model with thin-plate spline [28] or by fractional polynomials. An interaction of interest between age and years since first exposure (with age category and years since exposure as linear terms) was examined using the likelihood ratio test.

The adjusted prevalence of silicosis (predictive margin) [29] was calculated for each cluster using the fully adjusted logistic regression model fixing the value of all covariates to match the distribution in the overall sample. The 95% confidence interval (CI) associated with the adjusted values were calculated using the delta method [30]. Statistical analysis overall was performed using Stata Statistical Software version 14 (StataCorp, College Station, TX).

## Results

### Sample characteristics

**Fig. 1:**
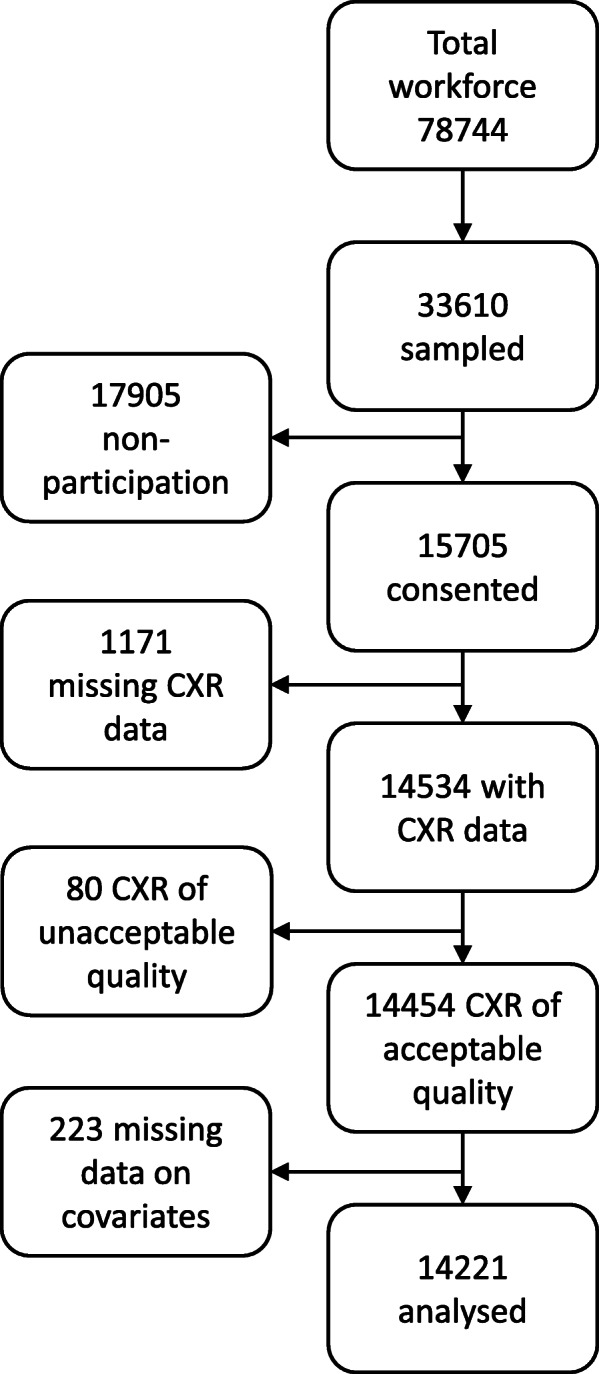
Thibela TB baseline survey sampling scheme. Footnotes: CXR: Chest radiograph Non-participation: Enrolment was a two-stage process – (i) miners randomly selected from the workforce were invited to attend the study centre post-shift, and (ii) those who attended were invited to participate in the study and were enrolled after giving informed consent. (Over 90% of those who attended in stage 2 consented)

Of the total sample recruited into the Thibela TB baseline survey, Fig. 1 details the subset included in this analysis, along with participation rates, radiographs read and number of participants with missing data.

The overall participation rate was 46.7%. The final sample for this analysis consisted of miners who had a radiograph reading and data on all the covariates of interest, for a total of 14,221 participants. The data did not show evidence of a difference in the prevalence of silicosis between the 233 willing participants with a missing value for at least one explanatory variable (and therefore excluded from the analysis) and those will a full set of values included in the analysis (*p* = 0.65).

Table 1 sets out the descriptive characteristics of the sample. Overall, 2.3% participants were female, 97.0% black, and 56.7% of South African nationality. The median age was 43 years (range 18–70 years) and the median number of years since starting in the industry was 19 (range 0–52 years). Regarding occupation, 93.1% were working full-time in an underground job and 8.7% were classified as skilled.

### Crude prevalences

**Fig. 2:**
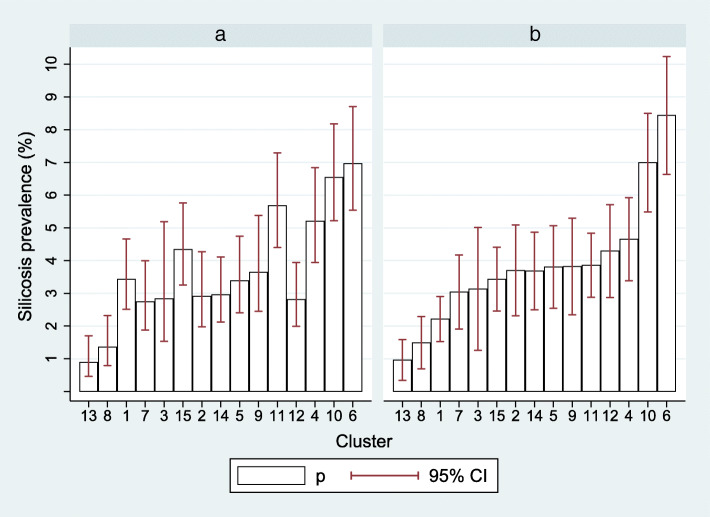
Silicosis prevalence by mine cluster, unadjusted (panel **a**) and adjusted (panel **b**) for covariates (*N* = 14,221). Footnote: Adjusted for gender, age, racial ascription, country of origin, duration since starting in the industry, skill level, and underground versus surface location. The figure shows predicted prevalences of silicosis in each cluster when the values of all other variables are fixed to match the distribution in the overall sample

At ILO grade > 1/1, the crude prevalence of silicosis, i.e. unadjusted for age or years since starting service, etc., was 3.8% (535/14221; 95% confidence interval (CI) 3.5–4.1%). A wide variation across clusters was found, with crude prevalences ranging from 0.9 to 7.0% (Table 1 and Fig. 2a). A modest variation was observed across regions (3.1 to 4.8%) and companies (2.6 to 4.4%). The prevalence of silicosis among those with more than 20 years since start of employment (49% of sample) was 6.8%, and among those aged 45 years or over (40% of sample) was 7.5%.

To examine the effect of a lower radiological threshold for silicosis diagnosis, Table 1 also reports the crude prevalence of silicosis defined by radiological profusion ILO grade ≥ 1/0. Overall silicosis prevalence at this threshold was 5.1% (730/14221, 95% CI 4.8–5.5%), increasing to 9.1% among those with 20 years since starting employment and 10.0% in those aged over 45 years. Crude prevalences across the different clusters ranged from 1.8 to 8.6%.

### Predictors of silicosis

**Fig. 3:**
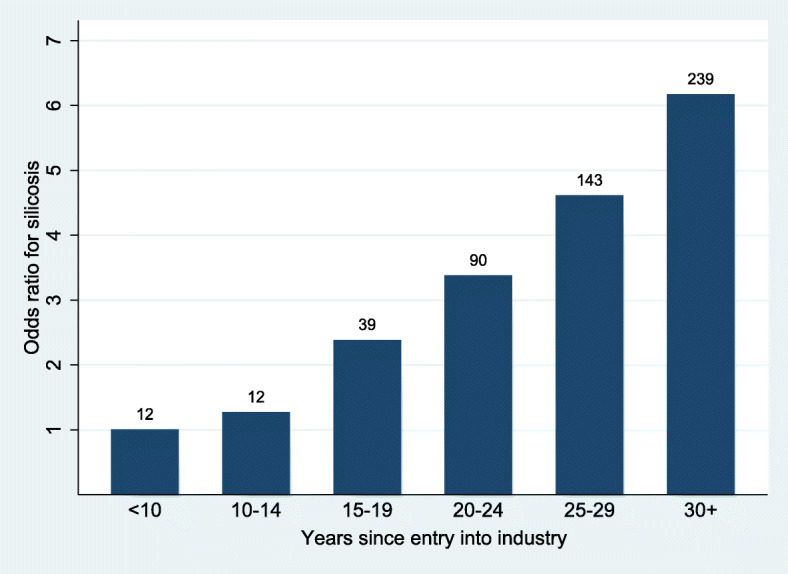
Relative odds of silicosis by years since first exposure, with silicosis counts, > 1/1 (*N* = 14,213). Footnote: Adjusted for gender, age, racial ascription, country of origin, duration since starting in the industry, skill level, underground versus surface location. Reference category: 0–4 years

Collinearity between age and years since first exposure was assessed by calculating the variance inflation factor, which in no case was higher than 6.5, below the common cut-off of 10. Both were therefore included in the model (Table 2, model A unless otherwise stated). Sharp positive gradients in the adjusted odds of silicosis were observed for both the effect of age, and years since starting in the industry (in both cases, p for linear trend < 0.001 in all models). The association by five-year increments of years since starting exposure, with the category 0–9 years as the reference, is illustrated in Fig. 3. Of those identified with silicosis, 95.5% had time from first exposure of 15 years or more. However no absolute exposure threshold was identified. There were 21 miners with < 10 years since first exposure identified with silicosis defined as > 1/0 (Table 2), all with ILO profusion 1/0 or 1/1 and none at higher grades (ILO 2 or 3) (data not shown). Conversely, all those with these more advanced grades (*n* = 57) had > 15 years of exposure. Modelling of years since first exposure as a continuous variable, either with the generalised additive model or by using fractional polynomials yielded no evidence of a threshold for the appearance of radiological silicosis (Additional File 1, Fig. S1 and S2). Finally, there was no interaction effect between age category and years since first exposure (logistic model, *p* = 0.45, see Additional File 1, Table S1).

**Table 2:** Predictors of silicosis (ILO grade > 1/1): unadjusted and adjusted models (*N* = 14,213)^a^

Exposure		Unadjusted model	Adjusted model A		Adjusted model B^b^	
		Odds Ratio (95% CI)	Odds Ratio (95% CI)	p	Odds Ratio (95% CI)	p
**Cluster**	1	1.0	1.0	< 0.001		
	2	0.84 (0.51; 1.41)	1.73 (1.03; 2.92)			
	3	0.82 (0.41; 1.66)	1.45 (0.70; 2.97)			
	4	1.55 (1.00; 2.38)	2.22 (1.42; 3.48)			
	5	0.99 (0.61; 1.59)	1.78 (1.09; 2.90)			
	6	2.11 (1.41; 3.15)	4.41 (2.91; 6.69)			
	7	0.79 (0.48; 1.32)	1.4 (0.83; 2.34)			
	8	0.39 (0.21; 0.73)	0.66 (0.35; 1.26)			
	9	1.07 (0.63; 1.79)	1.79 (1.05; 3.05)			
	10	1.97 (1.32; 2.94)	3.53 (2.34; 5.34)			
	11	1.70 (1.12; 2.57)	1.81 (1.18; 2.76)			
	12	0.81 (0.51; 1.31)	2.03 (1.24; 3.32)			
	13	0.25 (0.12; 0.52)	0.42 (0.20; 0.88)			
	14	0.86 (0.54; 1.37)	1.72 (1.06; 2.78)			
	15	1.28 (0.83; 1.98)	1.59 (1.02; 2.49)			
**Region**	Carletonville	1.0	–		1.0	0.035
	Orkney	1.30 (1.06; 1.6)	–		0.87 (0.68; 1.12)	
	Welkom	1.58 (1.28; 1.96)	–		1.43 (1.05; 1.93)	
**Company**	A	1.0	–		1.0	0.115
	B	0.64 (0.50; 0.81)	–		0.76 (0.54; 1.05)	
	C	1.10 (0.91; 1.33)	–		0.96 (0.69; 1.35)	
**Gender**	Male	1.0	1.0	0.729	1.0	0.674
	Female	0.15 (0.04; 0.62)	1.31 (0.3; 5.77)		1.39 (0.32; 6.11)	
**Age (years)**	18–34	1.0	1.0	< 0.001	1.0	< 0.001
	35–39	3.10 (1.38; 6.96)	1.73 (0.66; 4.55)		1.67 (0.64; 4.37)	
	40–44	9.16 (4.58; 18.36)	3.1 (1.17; 8.17)		2.92 (1.11; 7.65)	
	45–49	23.93 (12.23; 46.83)	5.77 (2.17; 15.38)		5.34 (2.01; 14.14)	
	50+	37.29 (19.15; 72.62)	7.25^c^ (2.68; 19.64)		6.5^c^ (2.41; 17.51)	
**Racial ascription**	Other	1.0	1.0	0.146	1.0	0.016
	Black	3.41 (1.40; 8.26)	2.81 (1.09; 7.21)		2.79 (1.09; 7.15)	
**Country of origin**^a^	South Africa	1.0	1.0	< 0.001	1.0	0.004
	Lesotho	1.72 (1.42; 2.08)	1.16 (0.95; 1.42)		1.16 (0.95; 1.42)	
	Swaziland	1.15 (0.73; 1.81)	0.84 (0.53; 1.34)		0.90 (0.57; 1.43)	
	Mozambique	0.72 (0.52; 1.00)	0.54 (0.38; 0.77)		0.61 (0.43; 0.86)	
	Botswana	2.3 (1.36; 3.88)	1.09 (0.63; 1.88)		1.06 (0.62; 1.82)	
**Years since entry into industry**	0–4	1.0	1.0	< 0.001	1.0	< 0.001
	5–9	2.54 (0.80; 8.04)	2.31 (0.71; 7.58)		2.20 (0.67; 7.19)	
	10–14	3.23 (1.13; 9.20)	2.04 (0.62; 6.71)		2.17 (0.66; 7.12)	
	15–19	9.39 (3.69; 23.88)	3.89 (1.2; 12.63)		4.19 (1.30; 13.51)	
	20–24	17.40 (7.06; 42.89)	5.53 (1.7; 17.98)		5.75 (1.78; 18.59)	
	25–29	33.52 (13.71; 81.93)	7.55 (2.3; 24.72)		7.93 (2.43; 25.82)	
	30+	52.06 (21.43; 126.47)	10.12^b^ (3.06; 33.41)		10.65^b^ (3.24; 34.99)	
**Skill level**	Skilled/Official	1.0	1.0	0.234	1.0	0.179
	Unskilled	1.76 (1.20; 2.58)	1.28 (0.84; 1.94)		1.26 (0.83; 1.92)	
**Under** **ground work**	None/part time	1.0	1.0	< 0.001	1.0	< 0.001
	Full time	0.52 (0.33; 0.81)	2.15 (1.35; 3.43)		2.14 (1.34; 3.42)	

p = Likelihood Ratio Test for exclusion of the relevant variable from the model

^a^Eight individuals from Namibia and Zimbabwe (“other” origin), none with silicosis, were excluded

^b^Shaft was replaced by region and company in Model B

^c^p for linear trend < 0.001 in both models

Black and underground miners respectively had higher silicosis odds than their comparison groups. The odds of silicosis varied by country of origin (*p* < 0.001). In particular, taking miners from South Africa as the reference, miners from Mozambique had a substantially lower odds of silicosis (OR 0.54; 95% CI 0.38–0.77).

Replacement of cluster with region or model did not change any of the associations. Both cluster (*p* < 0.0001) (model A) and, in a separate model (model B), region (*p* = 0.04), but not company (*p* = 0.12), were predictors of silicosis. Using cluster 1 as the reference category, the odds ratio (OR) for silicosis varied between 0.42 (95% CI 0.20–0.88) for cluster 13 to 4.41 (95% CI 2.91–6.69) for cluster 6. Of the regions, Welkom had higher odds than both Carltonville [OR 1.43; 95% CI 1.05–1.93) and Orkney.

Adjustment for tuberculosis had negligible impact on the estimates for cluster, company or region (data not shown). Running the same models with silicosis defined as ILO profusion grade ≥ 1/0 did not change the findings reported above regarding the association of odds of silicosis with cluster, region or company (data not shown).

### Adjusted prevalences across clusters

Figure 2, panels A and B, illustrates visually the effect of adjustment for all covariates (except for tuberculosis) on the difference in prevalence across the 15 clusters. The adjusted prevalence is that which would be observed in each cluster if the distribution of covariates matched the distribution in the overall sample (as shown in Table 1). On adjustment for covariates the interquartile range was halved, from 2.4 to 1.2%, with considerable reduction in variability across nine of the 15 clusters. However, the low prevalence in clusters 1,8 and 13 and high prevalence in clusters 10 and 6 persisted.

## Discussion

This study of over 14,500 miners adds to the previous analysis through an examination of heterogeneity in silicosis prevalence across mines shafts and of industry-wide predictors of silicosis. The crude prevalence of 3.8% at ILO profusion grade ≥ 1/1 is slightly lower than the crude prevalence of 4.1% reported in the previous analysis of this cohort (based on the same reader) which was conducted on a slightly smaller sample of miners and excluded some lower risk strata.[24].

The variation across the 15 individual clusters was reduced to some extent by adjusting for measured demographic and occupational covariates, but a wide range remained. The “unexplained” variation in silicosis prevalence between mine shafts/clusters may be due to real differences in silicosis risk, or residual confounding by correlated risks, specifically tuberculosis, including differences between mines in occupational medical or administrative practice. These are discussed below.

Regarding differences in true silicosis risk, although years since starting in the industry was controlled for in the analysis and had a very strong association with silicosis, this is a crude exposure metric. No dust concentration information was available. In particular, no information is publicly available on differences between shafts in airborne respirable crystalline silica dust concentrations, quartz content of dust, nor more generally mining conditions that might predispose to greater inhalation of free respirable silica in one shaft than another. The latter conditions include extent of development of new working areas, blasting practices such as re-entry times, ventilation systems and use of respiratory protection equipment. Differences have recently between found in in vitro toxicity of silica containing dust sampled from surface tailings storage facilities across the Witwatersrand [31]. However, the relevance to differences in inhaled dust underground in these mines is not yet known.

Strong disease related selection effects (health worker survivor effect) were reported in the previous analysis [24]. The probability of a miner remaining in service over the approximately 3 years of the Thibela TB study was halved if they had silicosis or tuberculosis at baseline. While HIV is a strong risk factor for tuberculosis the influence of differences in HIV infection prevalence across shafts could not be assessed. These selection effects could operate via work consequences of morbidity and associated administrative practices.

Given the close association of tuberculosis with silicosis [32], tuberculosis co-occurrence is likely to be a morbidity factor selecting miners with silicosis out of the industry [24]. Annual tuberculosis notification rates across shafts were extraordinarily high – ranging from around 2000 cases per 100,000 per annum to 8000 cases per 100,000 per annum - in the year prior to the survey [23]. Adjustment for tuberculosis using proxy variables (self-report and/or chest radiograph changes) in this analysis did not explain any of the variation in silicosis across shafts. However, some of the silicosis variation might be linked to unmeasured differences across clusters over time in the proportions of employees with severe or disabling tuberculosis, or those on treatment, particularly for drug resistant forms; for example, by choosing to return home rather than remaining in employment while being treated by mine medical services, or being offered or electing to take voluntary redundancy.

Other administrative factors influencing retention or departure of miners with silicosis include differential compliance with the Occupational Disease in Mines and Works Act (ODMWA) of 1993 [33], which prohibits miners certified with both silicosis and tuberculosis, defined as occupational disease in the second degree, from continuing in dusty work. Miners with one disease alone are required to be clinically assessed and reported under ODMWA for compensation claim purposes but are permitted to remain in dusty work [34]. However, such policies may be modified by the high rates of worker turnover in the setting of declining employment in the gold sector [35]. If different policies and practices are followed by different companies or even different mines within a company, a varying health worker survivor effect across shafts might ensue. This labour practice derived “healthy worker effect” could also be the result of differentially restrictive hiring practices regarding job applicants found to have silicosis.

It is of interest that age remained such a strong predictor of silicosis after taking into account years since commencing in the industry. Age effects are conventionally regarded as reflecting duration of exposure rather than being an independent predictor of occupational disease. However, our finding accords with the findings of a South African autopsy series [14] in which age remained a strong predictor of the presence of silicosis at autopsy *after* controlling for duration of employment. This association lacks explanation at this point, especially as no interaction with years of exposure was found (which would be the case if silica exposure at an older age were more pathogenic than at a younger age).

The exposure time response gradient was steep, as would be expected for radiological silicosis. It should be noted that the exposure metric was “years since starting in the industry” rather than duration of employment. The proportion of participants with interrupted service is unknown, but if there were many such, years since entering the industry would overestimate true exposure duration. Silicosis > profusion 1/1 was identified in a relatively small number of miners, none with advanced disease, with time since first exposure < 10 years, the figure cited as the exposure duration threshold between “accelerated” and chronic silicosis [36]. Short-service silicosis was thus uncommon in this cohort, but its continued detection suggests persistence of work practices or areas with uncontrolled intense exposure to silica.

The elevated risk among black miners and miners in full time underground employment is consistent with the higher occupational dust exposure associated with production work in gold mining. After adjusting for all covariates, company was no longer predictive of silicosis, but the Welkom region had an approximately 50% higher odds of silicosis than Carltonville and Orkney. This raises the question of difference in rock geochemistry and associated toxicity between different regions [31]. Mozambican miners had about half the odds of silicosis than South Africa origin miners after adjusting for covariates including years since first employment and skill category. Finally, the prevalence of silicosis among the relatively small subset of female miners was low. However, the number of women recruited to underground work has increased in recent years [37] and recent autopsy studies have demonstrated silicosis in female gold miners [38].

### Limitations

The previous silicosis publication on this cohort provides a detailed analysis of both potential selection bias and information bias, specifically radiological misclassification [24]. It is unlikely that miners’ choice to participate in the Thibela TB baseline survey was linked to their baseline silicosis status. The original Thibela TB study readings were used as it was not possible to re-read over 14,500 radiographs. However, a validation sub-study was undertaken in which an experienced occupational medicine physician (“second reader”) read all radiographs reported as silicosis ILO grade ≥ 0/1 by the primary reader, and a random sample of those reported as completely normal (25 per cluster), for a total of 991. The primary reader provided high sensitivity (92%) but only modest specificity (58%). Extrapolation of these effects to the whole study sample indicated a likely underestimate of the prevalence of silicosis by the primary reader [24]. However, misclassification of silicosis is likely to have been non-differential with respect to the important covariates such as age, shaft, company, region or the exposure variables. The effect would therefore be to under- rather than overestimate associations between exposure and silicosis.

With regard to reducing exposure misclassification, the only other exposure variable available in the Thibela TB study was current occupation. It was not possible to construct an exposure history to allow for various occupations a miner might have held over a working life. A large number of occupational terms were used by respondents in interviews, suggesting multiple terms in use for the same occupation across different shafts, and it was also not possible to reduce this heterogeneity into a manageable number that might have allowed an independent ranking by exposure. Information on dust exposure or use of respiratory protection, that would have allowed comparisons, whether across shafts are across occupations, were not available. However, a summary of a large number of measurements of respirable silica on 48 South African gold mines between 1995 and 1997, a decade before this study was conducted, reported that 43% of mines had a high proportion of their measurements between 0.1 mg/m^3^, the current South African mining occupational exposure limit, and 0.4 mg/m^3^; and a further 40% had most of their measurements above 0.4 mg/m^3^ [39]. Similarly, in a 2007/8 survey of a sample of 209 former gold miners mostly from high dust occupations, only 20.9% listed dust masks as one of the means used to minimise dust underground [40].

Like most of the studies of silicosis in the South African mining industry, this analysis is restricted to working miners. Knowledge of the silicosis experience of the large population of black ex-miners, most of who are migrants from rural areas of Southern Africa, is limited [8, 9, 12, 41]. Factors affecting the progression, radiological incidence and prevalence of silicosis in ex-miners include high initial mortality rates [42] and the poorly understood effects of latency from retained dust load and sub-radiological silicosis [43], both of which may result in the first appearance of radiological appearance after exposure has ended. In studies of former miners, much higher prevalences of silicosis with relatively short duration of employment have been found [8, 9].

High HIV prevalence recorded in migrant miners at a time [12] when coverage of antiretroviral therapy was low, along with ongoing risk of tuberculosis (including recurrent disease) due to HIV and dust, would further shape survival and the associated silicosis prevalence. Health system factors contributing to deficient understanding of the epidemiology of silicosis in ex-miners include lack of surveillance and recognition of silicosis once miners have left the mine [40, 41], and relative absence of ex-miners from official autopsy and compensation statistics [44].

## Conclusion

This analysis has revealed several features of silicosis prevalence in the South African industry with practical implications. Almost all the miners with silicosis, and all those with ILO profusion grade > 2, had time from first exposure of 15 years or more. However, there was no absolute threshold, indicating the presence of short-service silicosis even in working miners.

The study confirms known predictors of radiological silicosis of years since first employment, underground work and racial ascription (a proxy for dusty occupations). Differences between shafts could not be fully explained by the covariates measured in this study, while company was not a predictor. Age was a strong predictor, a consideration which needs to be taken into account in “pre-screening” former mineworkers for likelihood of silicosis in current large-scale medical assessment programmes.

As this study suffers from the typical limitation of “snapshot” surveys of working miners across the industry in a one-time window (2004–2009), with the associated healthy worker effect [24], two long-neglected activities are needed. The first is a widening of the scope of surveillance and research to include ex-mineworkers. Studies of ex-miners, particularly cohort studies of black miners, are needed to understand latency, progression and tuberculosis onset and recurrence phenomena.

The second is more accurate record keeping for individual miners – lifelong mining history including occupations and dust exposure indicators, i.e. concentrations and quartz fractions (if possible) to allow cumulative silica exposures to be computed; as well as exit examination information for silicosis and tuberculosis status. The Mine and Health Safety Act requires medical records to be kept for 40 years [45]. Efforts should be made to keep these records accessible, as in the electronic database of a group of gold mining companies currently under compilation (Dr. Zahan Eloff – personal communication), to enable linkage with later medical assessment. This is urgent as individual companies’ close operations [46] and place their records in storage.

For public accountability, company reporting of annual silicosis prevalence and incidence rates, and silica dust concentrations, in the same way as annual accident and injury data are reported should be required. These should be augmented by autopsy data and recording of accepted silicosis claims (by company of longest service) by the statutory compensation agency, and the recently formed Tshiamiso Trust. Independent epidemiological and occupational hygiene assessment (analogous to financial auditing) is essential for the integrity of the process.

Employment on the gold mining industry in South Africa is declining and the changes in the industry are likely to be rapid in the current uncertain political and economic climate. There will be strong pressures to relegate the silicosis problem to a “legacy” effect fully dealt with by the settlement of recent litigation, and one not requiring further research or public attention. This would be a dereliction of responsibility to both current and former mine workers.

## Supplementary information

**Additional file 1: Figure S1.** Relationship between years in the industry and risk of silicosis: generalised additive model. **Figure S2.** Relationship between years in the industry and risk of silicosis: Fractional polynomial model. **Table S1.** Logistic model for prediction of silicosis including interaction between age category and years since first exposure. Estimated coefficients.

## Data Availability

The data that support the findings of this study are not publicly accessible. Further interested inquiry should be made to the study Principle Investigator, Prof Gavin Churchyard, corresponding author with permission from Dr. Dave Knight.

## Abbreviations

ILO: International Labour Organisation
CI: Confidence interval
aOR: Adjusted odds ratio
SADC: Southern African Development Community
HIV: Human immunodeficiency virus
ODMWA: Occupational Disease in Mines and Works Act

## Footnotes

1 Mining operations are organised around one or more shafts, each a single surface excavation with accompanying equipment and underground excavations. Miners are accommodated in company hostels, usually one per shaft, but in this case there was one hostel serving two shafts. In the TB prevention trial on which this study is based, the term “cluster” was used to denote all the miners living in a hostel (*n* = 15), serving 16 mine shafts.
