# Supplementary material for: Predictors of silicosis and variation in prevalence across mines among employed gold miners in South Africa
Source: BMC Public Health. 2020 Jun 1;20:829. doi: 10.1186/s12889-020-08876-2 (PMC7268682; doi:10.1186/s12889-020-08876-2)
Supplement: Supplementary file 1 — Additional file 1: Figure S1. Relationship between years in the industry and risk of silicosis: generalised additive model. Figure S2. Relationship between years in the industry and risk of silicosis: Fractional polynomial model. Table S1. Logistic model for prediction of silicosis including interaction between age category and years since first exposure. Estimated coefficients. [file 12889_2020_8876_MOESM1_ESM.pdf]

Predictors of silicosis and variation in prevalence across mines among employed gold miners in South Africa.

Knight D, Ehrlich R, Cois A, Fielding K, Grant AD, Churchyard G

Additional File 1

Figure S1: Relationship between years in the industry and risk of silicosis: generalised additive model*.

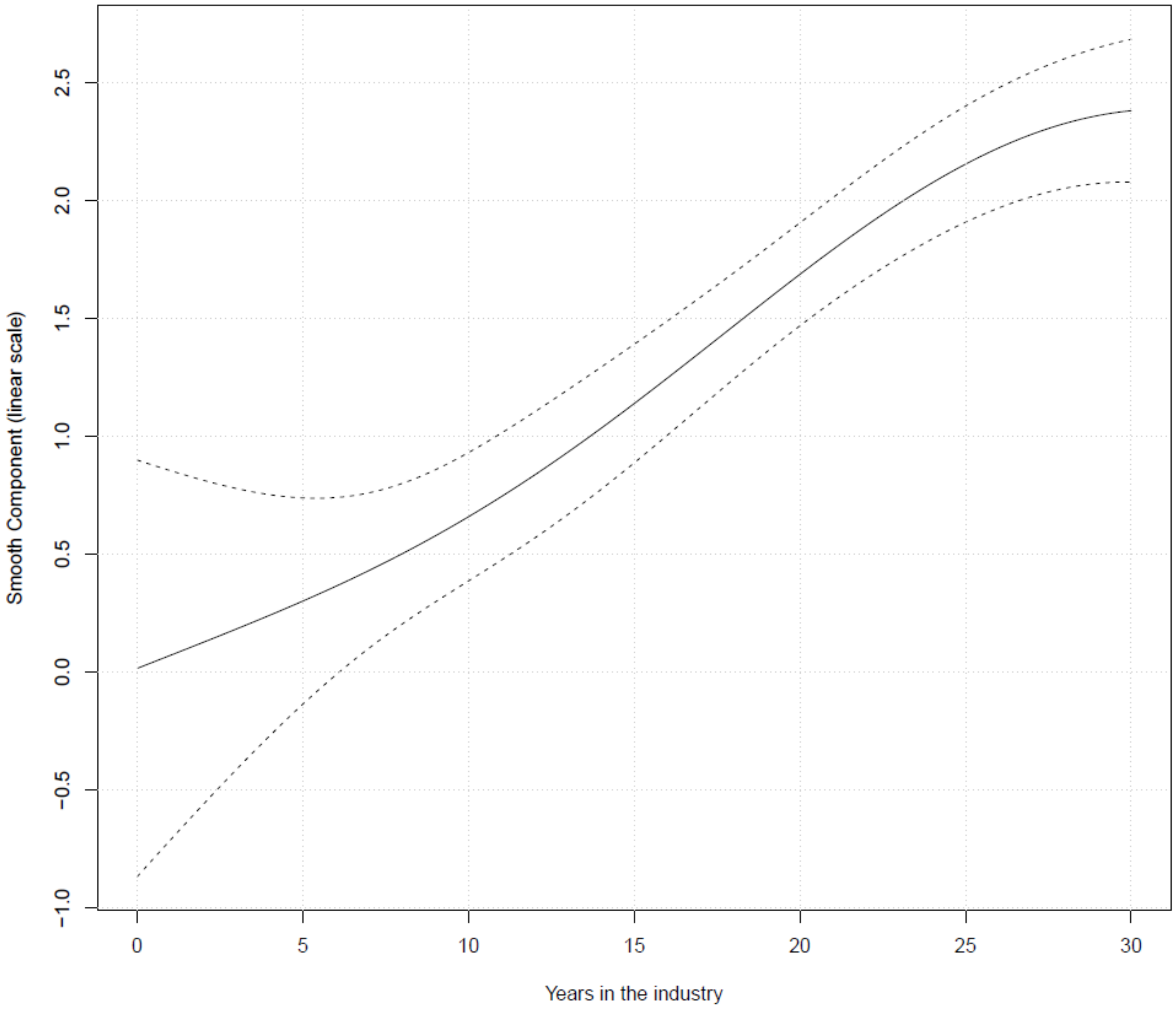

* Thin plate spline with K=5. Output centred so that risk=0 for 0 years in the industry.

Figure S2: Relationship between years in the industry and risk of silicosis: Fractional polynomial model*.

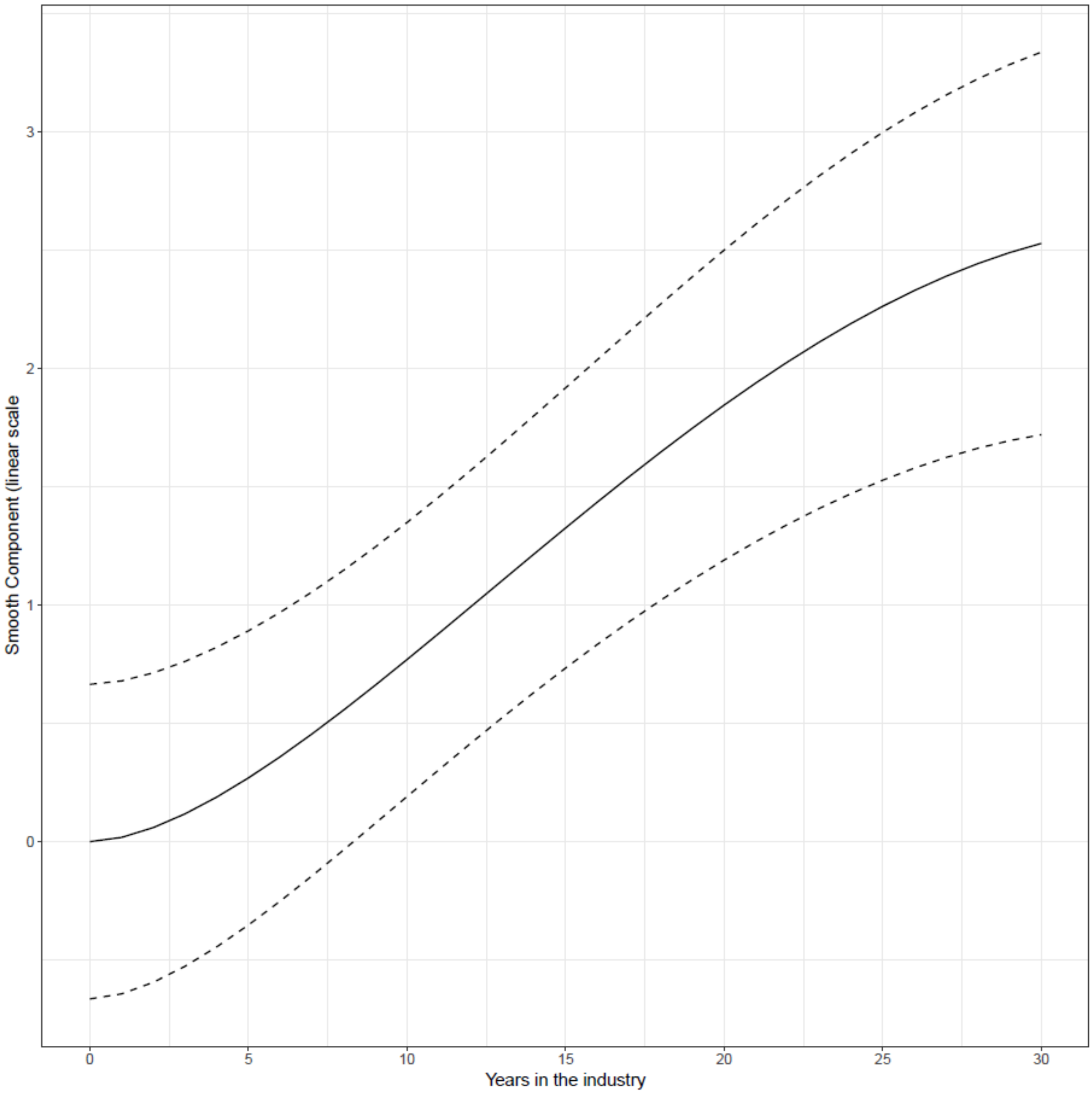

* Stata default values for powers to be searched (-2, -1, -0.5, 0 .5, 1, 2, 3) and maximum degree of the polynomial (2). Plotted output centred so that risk=0 for 0 years in the industry.

Table S1: Logistic model for prediction of silicosis including interaction between age category and years since first exposure. Estimated coefficients.

silibin11	Odds Ratio	Std. Err.	z	P>|z|	[95% Conf.	Interval]
cluster						
Beatrix 1 2 3	1.704018	.4549232	2.00	0.046	1.009783	2.875545
Beatrix 4	1.455404	.5346897	1.02	0.307	.7083814	2.990198
Cooke 3	2.210243	.5045339	3.47	0.001	1.412978	3.457359
Elandsrand	1.751467	.4358742	2.25	0.024	1.0754	2.852555
GN MK	4.334266	.9234137	6.88	0.000	2.854746	6.580573
Kloof 1 3 4	1.370326	.3621594	1.19	0.233	.8163234	2.300305
Kloof 7	.6495908	.2140216	-1.31	0.190	.3405598	1.239043
Kloof 8	1.758951	.4797976	2.07	0.038	1.030546	3.002203
Kopanang	3.478614	.7340283	5.91	0.000	2.300343	5.260412
Masimong 5	1.793757	.3875451	2.70	0.007	1.174518	2.739476
Mponeng	1.962093	.4927286	2.68	0.007	1.199396	3.209791
Tau Lekoa	.4150288	.1559488	-2.34	0.019	.1987174	.8668036
Tau Tona	1.697019	.4169042	2.15	0.031	1.048511	2.74663
Tshepong	1.576502	.358099	2.00	0.045	1.010058	2.460612
sex						
female	1.129782	.8606711	0.16	0.873	.2538322	5.028546
agecat						
35-39	4.699053	4.332858	1.68	0.093	.7711564	28.63374
40-44	3.629001	4.467364	1.05	0.295	.3250446	40.51643
45-49	5.41092	6.448902	1.42	0.157	.5233384	55.94478
50+	5.844591	6.867847	1.50	0.133	.5841527	58.47657
yindustrycat5						
5-9	2.277851	1.871359	1.00	0.316	.4552232	11.39794
10-14	3.889701	3.205762	1.65	0.099	.7733642	19.56358
15-19	12.51139	17.1688	1.84	0.066	.8496525	184.234
20-24	12.1145	16.21811	1.86	0.062	.8785617	167.0471
25-29	8.696588	11.55712	1.63	0.104	.6429326	117.6339
30+	13.85319	18.25892	1.99	0.046	1.046261	183.4253
agecat#yindustrycat5						
35-39#5-9	.4963554	.6447504	-0.54	0.590	.0389142	6.331072
35-39#10-14	.1913051	.2294855	-1.38	0.168	.018224	2.008212
35-39#15-19	.128067	.2030509	-1.30	0.195	.005726	2.864324
35-39#20-24	.1084553	.1970858	-1.22	0.222	.0030792	3.819972
40-44#5-9	2.272314	3.49727	0.53	0.594	.1112762	46.40176
40-44#10-14	.150768	.2228048	-1.28	0.200	.0083254	2.730324
40-44#15-19	.224298	.1362143	-2.46	0.014	.0682178	.7374848
40-44#20-24	.4929016	.232623	-1.50	0.134	.1954515	1.24303
45-49#10-14	1.262533	1.511633	0.19	0.846	.1208084	13.19435
45-49#15-19	.4359575	.2404643	-1.51	0.132	.1478906	1.285132
45-49#20-24	.397186	.1490739	-2.46	0.014	.1903341	.8288411
45-49#25-29	1.090098	.321835	0.29	0.770	.6111689	1.94433
black						
Other	.3506415	.1685609	-2.18	0.029	.1366697	.89961
corigindef						
Lesotho	1.189335	.1243603	1.66	0.097	.9689475	1.459851
Swaziland	.8579425	.2050602	-0.64	0.521	.5370432	1.370589
Mozambique	.5292937	.0941498	-3.58	0.000	.3734966	.7500787
Botswana	1.101456	.3099554	0.34	0.731	.6345043	1.912052
occlevl						
Skilled	.7971475	.1700487	-1.06	0.288	.5247581	1.210928
undergroundFT						
None/Part time	.4630834	.1106439	-3.22	0.001	.289923	.7396661

Notes

- 1.agecat#4.yindustrycat5, 1.agecat#5.yindustrycat5, 1.agecat#6.yindustrycat5, 2.agecat#5.yindustrycat5 and 2.agecat#6.yindustrycat5 identify no observations in the sample;

- 3.agecat#5.yindustrycat5, 4.agecat#6.yindustrycat5, 5.agecat#2.yindustrycat5, 5.agecat#3.yindustrycat5, 5.agecat#4.yindustrycat5, 5.agecat#5.yindustrycat5 and 5.agecat#6.yindustrycat5 omitted because of collinearity;
- 8.corigindef != 0, 1.agecat#3.yindustrycat5 != 0, 1.agecat#3.yindustrycat5 != 0, 3.agecat#0.yindustrycat5 != 0, 3.agecat#6.yindustrycat5 != 0, 4.agecat#0.yindustrycat5 != 0, 4.agecat#1.yindustrycat5 != 0, 5.agecat#0.yindustrycat5 != 0, 5.agecat#1.yindustrycat5 predict failure perfectly and are dropped from the model.

Likelihood ratio test
for significant differences in model fit between this model and the base model without interaction between age category and years since first exposure

Base model: log-likelihood = -1927.9448
Interaction model: log-likelihood = -1915.942

LR test statistics: -2*(-1927.94+1915.94) = 24.00, df = 24 , p-value = 0.45
